# Erythro-VLPs: Anchoring SARS-CoV-2 spike proteins in erythrocyte liposomes

**DOI:** 10.1371/journal.pone.0263671

**Published:** 2022-03-11

**Authors:** Sebastian Himbert, Isabella Passos Gastaldo, Rashik Ahmed, Karla Martinez Pomier, Braeden Cowbrough, Dushyant Jahagirdar, Samantha Ros, Janos Juhasz, Harald D. H. Stöver, Joaquin Ortega, Giuseppe Melacini, Dawn M. E. Bowdish, Maikel C. Rheinstädter

**Affiliations:** 1 Department of Physics and Astronomy, McMaster University, Hamilton, ON, Canada; 2 Origins Institute, McMaster University, Hamilton, ON, Canada; 3 Department of Chemistry and Chemical Biology, McMaster University, Hamilton, ON, Canada; 4 Department of Biochemistry and Biomedical Sciences, McMaster University, Hamilton ON, Canada; 5 Department of Medicine, McMaster University, Hamilton, ON, Canada; 6 McMaster Immunology Research Centre, McMaster University, Hamilton, ON, Canada; 7 Firestone Institute for Respiratory Health, St. Joseph’s Healthcare, Hamilton, ON, Canada; 8 Department of Anatomy and Cell Biology, McGill University, Montreal, QC, Canada; 9 Juravinski Cancer Centre, Department of Medical Physics, Hamilton, ON, Canada; National Institute of Child Health and Human Development, UNITED STATES

## Abstract

Novel therapeutic strategies are needed to control the SARS-CoV-2 (severe acute respiratory syndrome coronavirus 2) pandemic. Here, we present a protocol to anchor the SARS-CoV-2 spike (S-)protein in the cytoplasmic membranes of erythrocyte liposomes. A surfactant was used to stabilize the S-protein’s structure in the aqueous environment before insertion and to facilitate reconstitution of the S-proteins in the erythrocyte membranes. The insertion process was studied using coarse grained Molecular Dynamics (MD) simulations. Liposome formation and S-protein anchoring was studied by dynamic light scattering (DLS), ELV-protein co-sedimentation assays, fluorescent microcopy and cryo-TEM. The Erythro-VLPs (erythrocyte based virus like particles) have a well defined size of ∼200 nm and an average protein density on the outer membrane of up to ∼300 proteins/*μ*m^2^. The correct insertion and functional conformation of the S-proteins was verified by dose-dependent binding to ACE-2 (angiotensin converting enzyme 2) in biolayer interferometry (BLI) assays. Seroconversion was observed in a pilot mouse trial after 14 days when administered intravenously, based on enzyme-linked immunosorbent assays (ELISA). This red blood cell based platform can open novel possibilities for therapeutics for the coronavirus disease (COVID-19) including variants, and other viruses in the future.

## Introduction

The outbreak of the coronavirus disease 19 (COVID-19) has challenged and still challenges the world in an unprecedented manner. It has led to over 374 million infections and more than 5,600,000 deaths globally [1] (as of January 30, 2022). The adverse effects of this global crisis, which has permeated all aspects of day-to-day living, including personal life, economy, and health care systems, substantiates an urgent need for novel diagnostics, therapeutics and vaccines.

The severe acute respiratory syndrome-coronavirus-2 (SARS-CoV-2) is mainly transmitted via respiratory droplets [2, 3]. In the lung, both SARS-CoV-2, as well as its precursor SARS-CoV, primarily infect the ciliated bronchial epithelial cells and type 2 pneumocytes [4–6] through the angiotensin converting enzyme 2 (ACE-2). This triggers a cascade of reactions leading to the fusion of the virus with the host cell and its reproduction, ultimately causing COVID-19.

SARS-CoV-2 is an enveloped, single and positive stranded RNA virus [4, 7]. Of the three protein components on the viral envelope, the spike (S-)protein binds to the human ACE-2 receptor with a high affinity [7–10], and catalyzes the viral and host membrane fusion to initiate the infection [10, 11]. It is a densely glycosylated transmembrane protein that forms the characteristic surface spikes of the corona virus [10]. The protein also induces neutralizing antibody and T-cell responses, and is, therefore, an important target for vaccine development [12]. The structure and conformations of the SARS-CoV-2 S-protein have been elucidated, however, this is still a highly active field of research [7, 9, 11]. The basic structure consists of an ectodomain trimer that includes the receptor binding domain (RBD), a trans-membrane domain (TMD), and a cytoplasmic domain (CPD).

The development of diagnostics, therapeutics and vaccines for SARS-CoV-2 challenges our current nanomedical manufacturing capabilities. Several SARS-CoV-2 vaccines have been developed [13, 14]. Gene-based vaccines deliver gene sequences that encode protein antigens that are produced by host cells. These include recombinant vaccine vectors (including AstraZeneca, Johnson & Johnson), or nucleic acid vaccines (including Pfizer/BioNTech, Moderna) [15]. The mRNA vaccines have shown a high potency [16] and typically require carriers, such as nanoparticles, as mRNA is quickly degraded by cellular processes.

Here, we present an alternative approach to administer the S-protein using endogenous carriers by the *in-vitro* functionalization of red blood cells (RBCs) through directly anchoring the SARS-CoV-2 S-protein into the RBCs’ cytoplasmic membrane. Nanocarriers adsorbed on RBCs have been shown to improve delivery for a wide range of carriers and viral vectors [17, 18] and their biocompatibility may be advantageous over synthetic carriers [19, 20]. However, their potential for therapeutic applications, such as drug delivery [21, 22] and immunological functions [23–26] has been started to be exploited only recently. RBCs have been reported previously to catch immune complexes and bacteria and present them to Kupffer cells in the liver and Antigen-Presenting Cells (APCs) in the spleen [27, 28]. Through this mechanism, virus like particles prepared using RBCs (Erythro-VLPs) can potentially lead to antibody production, higher central memory T cell, and lower regulatory T cell response [29] when delivered to the spleen. As will be shown below, these Erythro-VLPs exhibit dose-dependent binding to ACE-2 in biolayer interferometry assays and seroconversion in a pilot mouse trial as confirmed in enzyme-linked immunosorbent assays.

## Results & discussion

**Fig 1:**
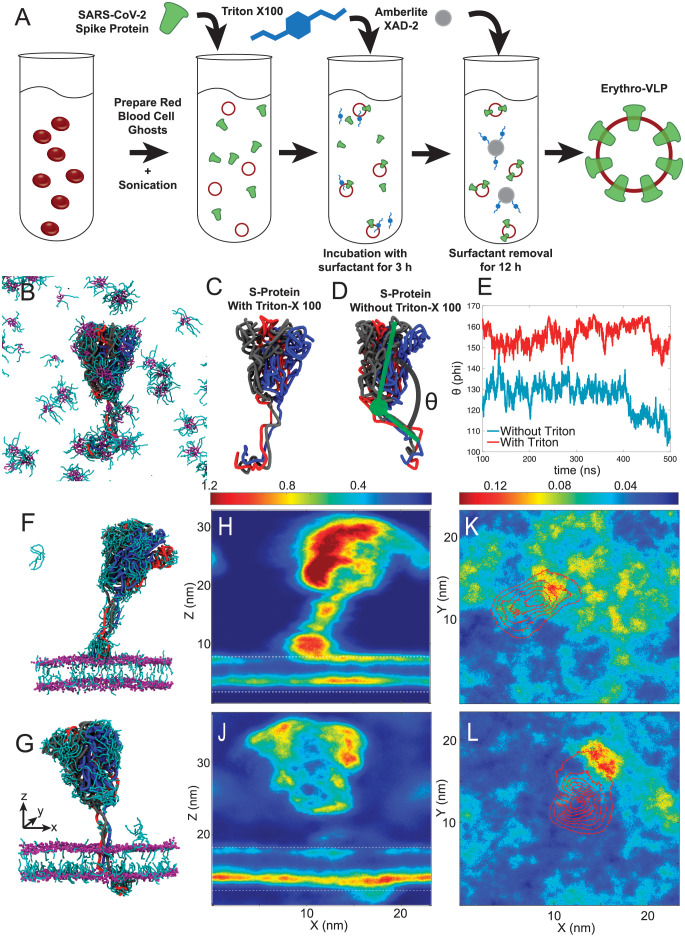
**A**: Preparation protocol for Erythro-VLPs: Erythrocyte liposomes were prepared from human RBCs. 14 mg/ml erythrocyte liposomes were incubated in a 3 *μ*M and 25 mM Triton-X100 solution. The detergent was removed by Amberlite XAD-2 and size-exclusion chromatography (SEC). **B**: Snapshot of a MD simulation of the S-protein in a 25 mM aqueous Triton-X 100 solution after 500 ns. The three chains of the protein are visualized as black, red and blue tubes; Triton-X 100 is represented by cyan rods (hydrophilic head group) and purple rods (hydrocarbon tails). **C** and **D**: Snapshots of the S-protein in aqueous solution after 500 ns with and without Triton-X 100, respectively. The angle Θ measures the tilt of the TMD relative to the ectodomain trimer and is plotted in **E**. **F** MD snapshot after 50 ns of the S-protein insertion process into the erythrocyte membrane. **G** Snapshot after 500 ns, with the S-protein fully embedded in the membrane. Triton-X 100 density maps from both simulations averaged along the *y*-axis are displayed in **H** and **J**, maps averaged along the *z*-axis in **K** and **L**.

Erythro-VLPs were produced as sketched in Fig 1A. Briefly, erythrocyte liposomes were prepared as detailed in [30], and incubated with a 3 *μ*M S-protein solution. A surfactant (Triton-X 100) was used to reconstitute the S-protein (at a concentration of 25 mM). The surfactant was then removed by Amberlite XAD-2 beads and subsequent size-exclusion chromatography (SEC).

The transport of the the CPD across the hydrophobic membrane core is essential to anchor the S-protein in the membrane of the erythrocyte liposomes. Surfactants are often used to facilitate these insertion processes [31–33]. An all-atom Molecular Dynamics model of the full length S-protein has been previously published by [34] and was used to generate coarse grained MD simulations in an aqueous solution with and without 25 mM Triton-X 100, as described in the *Materials & Methods*. Three-dimensional renders of the simulation are depicted in Fig 1B–1D. Triton-X 100 micelles were observed in the simulations in Fig 1B, and the surfactant was found to bind to the protein, particularly to the TMD and CPD. While the hydrophobic hydrocarbon moiety (tail) of Triton-X 100 was found to associate with the TMD, likely to shield its hydrophobic core region from the aqueous environment, the hydrophilic head groups preferably interacted with the CPD. The angle Θ (in Fig 1D) measures the tilt of the membrane domain relative to the protein’s ectodomain trimer. Fig 1E shows the time-behavior of this tilt-angle for simulations with and without Triton-X 100. An average angle of Θ = 155±5° was determined in the presence of Triton-X 100 while the angle was significantly reduced (Θ = 126±7°) in the absence of this surfactant (Fig 1C and 1D). It can be speculated that the straight orientation of the TMD facilitates membrane entry, in contrast to the tilted structure in the absence of the surfactant.

MD snaphots of the S-protein’s insertion process are shown in Fig 1F and 1G. Triton-X density maps, averaged along the *y*-axis, are depicted in Fig 1H and 1J (the membrane is indicated by white dotted lines). Lateral Triton-X distribution within the membrane, averaged along the *z*-direction, is shown in Fig 1K and 1L. When the S-protein is close to the membrane (in Fig 1F, 1H and 1K), the CPD is the first point of contact. A high surfactant density is observed around the CPD, which likely facilitates insertion and passage through the membrane by lowering the hydrophobic mismatch between CPD and hydrophobic membrane core. The intra-membrane surfactant density is also increased in both leaflets, around the protein’s lateral location. Once the protein is fully anchored (Fig 1G, 1J and 1L), surfactant density around the TMD is significantly reduced and remains concentrated around the CPD and the surrounding inner leaflet. This leaflet was observed to have a slightly larger area per lipid of 0.54 nm^2^, as compared to 0.52 nm^2^ in the outer leaflet, likely due to an asymmetric lipid distribution and a slightly higher density of polyunsaturated lipid tails in the inner leaflet. The additional space is likely responsible for the asymmetric distribution of Triton-X 100 between both leaflets.

**Fig 2:**
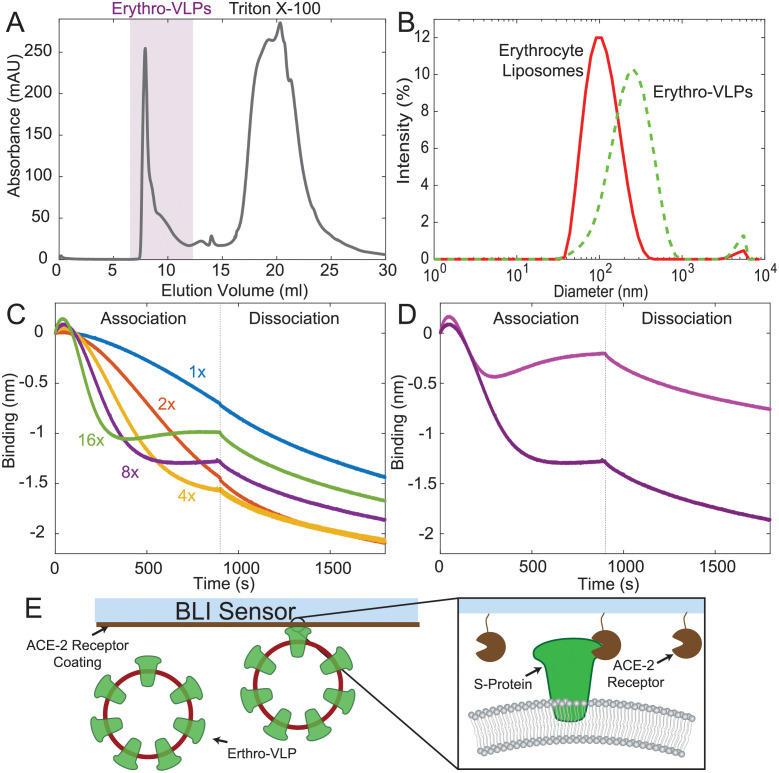
**A**: Size exclusion chromatogram of the Erythro-VLPs showing two signals from Erythro-VLPs and Triton-X100. **B**: Size distribution of Erythro-VLPs, as determined by DLS. While erythrocyte liposomes measured 102 nm (polydispersity: 0.19) an average diameter of 222 nm (polydispersity: 0.32) was determined for the S-protein carrying liposomes. **C**: Binding of Erythro-VLPs to human ACE-2 protein was measured by biolayer interferometry (BLI). A dose-dependent reduction in BLI signal was observed upon exposure of the ACE-2 immobilized biosensors to increasing concentrations of Erythro-VLPs, consistent with the binding of large particles to the optical biosensor. **D**: Association and dissociation curves for Erythro-VLPs in the absence (light purple) and presence (dark purple) of human ACE-2 immobilized onto the biosensor. The dark purple curve is reproduced from C (8×) for the purpose of comparison. **E**: Schematic of the BLI. Biotinylated human ACE-2 was immobilized onto the Streptavidin BLI sensor. The sensor was then exposed to Erythro-VLPs and association and dissociation was monitored.

The Erythro-VLPs were purified using SEC, shown in Fig 2A, detected between an elution volume of 7 ml and 12 ml. Remaining Triton-X 100 micelles were detected between an elution volume of 15 ml and 30 ml, showing that the Amberlite XAD-2 beads do not completely remove Triton-X 100 and subsequent separation with SEC is essential for the purification of the Erythro-VLPs. The size distribution of the purified erythrocyte liposomes with and without S-protein was determined by dynamic light scattering (DLS) and is shown in Fig 2B. While erythrocyte liposomes measured 102 nm (polydispersity: 0.19), in good agreement with previous results [35], an average diameter of 222 nm (polydispersity: 0.32) was determined for the Erythro-VLPs.

The concentration of proteins on the liposomes can be estimated using the following assumptions: 70% of the RBC’s cytoplasmic membrane’s mass are known to be lipids [30], with an average molecular mass of 700 g/mol. A liposome with a diameter of 100 nm has a surface area of ∼126,000 nm^2^. Assuming a typical area per lipid of 0.6 nm^2^ in each leaflet, each liposome contains ∼42,000 lipid molecules. Cholesterol is well known to affect the area per lipid in a multi-component plasma membrane [36] and thus may effect this calculation. The RBC’s cytoplasmic membrane is composed of approximately 50 mol% cholesterol which can be assumed to have an area per molecule of 0.4 nm^2^ [36] resulting in an average area per lipid of (0.6 nm^2^ + 0.4 nm^2^)/2 = 0.5 nm^2^. This increases the estimated number of lipid molecules per vesicle to ∼51,000. An initial concentration of erythrocyte liposomes of 14 mg/ml then corresponds to a liposome concentration of ∼30 nM. A loading efficiency of 40% was determined in ELV-protein co-sedimentation assays (shown in S2 Fig). The average number of proteins per liposome is then calculated by dividing the molar concentration of proteins in solution (3 *μ*M) by the liposome concentration to ∼36 proteins/Erythro-VLP (on average). This corresponds to an average protein density of ∼300 proteins/*μ*m^2^.

Binding to ACE-2 was confirmed through biolayer interferometry (BLI) [37] (sketched in Fig 2E). When incorrectly embedded, the S-protein can not bind to the ACE-2 receptor. This assay is thus important to assess the correct functional conformation of the S-protein in the membrane-embedded state. A dose-dependent reduction in BLI signal was observed upon exposure of the ACE-2 immobilized biosensors to increasing concentrations of Erythro-VLPs, consistent with the binding of large particles to the optical biosensor (Fig 2C). Interestingly, addition of higher concentrations of Erythro-VLPs (>4×) resulted in more prominent binding at earlier time points which saturates at smaller negative λ values. This observation is consistent with a concentration-dependent clustering of Erythro-VLPs, which when bound to the sensor chip sterically hinder [38] binding of further Erythro-VLPs. To exclude the possibility of additional binding contributions from the erythrocyte liposomes, we titrated erythrocyte liposomes lacking the S-protein to the ACE-2 immobilized sensor chip. Unlike the binding of the Erythro-VLPs, addition of erythrocyte liposomes to ACE-2 resulted in a positive wavelength change (Δλ) (see S3 Fig). This observation not only suggests that the binding of Erythro-VLPs to ACE-2 is distinct in terms of the observed wavelength change, as compared to the erythrocyte liposomes, but also implies that the saturation of BLI signal at lower negative λ values observed at higher Erythro-VLP concentrations could arise from weaker binding contributions from erythrocyte liposomes.

Notably, in the absence of ACE-2 conjugation to the biosensor, the wavelength change due to binding is significantly diminished, suggesting that the S-protein—ACE-2 interaction is the predominant source of binding probed through BLI (Fig 2D). Overall, these findings indicate that the S-protein’s RBD recognizing human ACE-2 remains solvent-exposed after embedding into the erythrocyte liposomes (Fig 2E). While it can not be excluded that that the S-protein embeds in two orientations, with the RBD domain facing outwards or inwards the erythrocyte liposomes, the observation of a positive binding affinity of the Erythro-VLPs (as well as the positive immune response in mouse models further below) confirms that a significant fraction of S-proteins are facing outwards and remain in an active conformation.

Giant unilamellar vesicles (GUVs) were prepared to visualize the partitioning of the S-proteins in the erythrocyte liposomes. While electroformation is commonly used to fabricate GUVs, this method is difficult in physiological buffers because of electrolysis and the corresponding gas formation in the presence of salts [31, 32]. Giant Erythro-VLPs were, therefore, prepared using gel-assisted swelling where the Erythro-VLPs were first dried on an agarose gel. Giant Erythro-VLPs then formed spontaneously when the gel-liposome film was rehydrated. The procedure is known to lead to a homogeneous protein distribution among the liposomes and allows a rapid preparation in physiological buffers [31].

**Fig 3:**
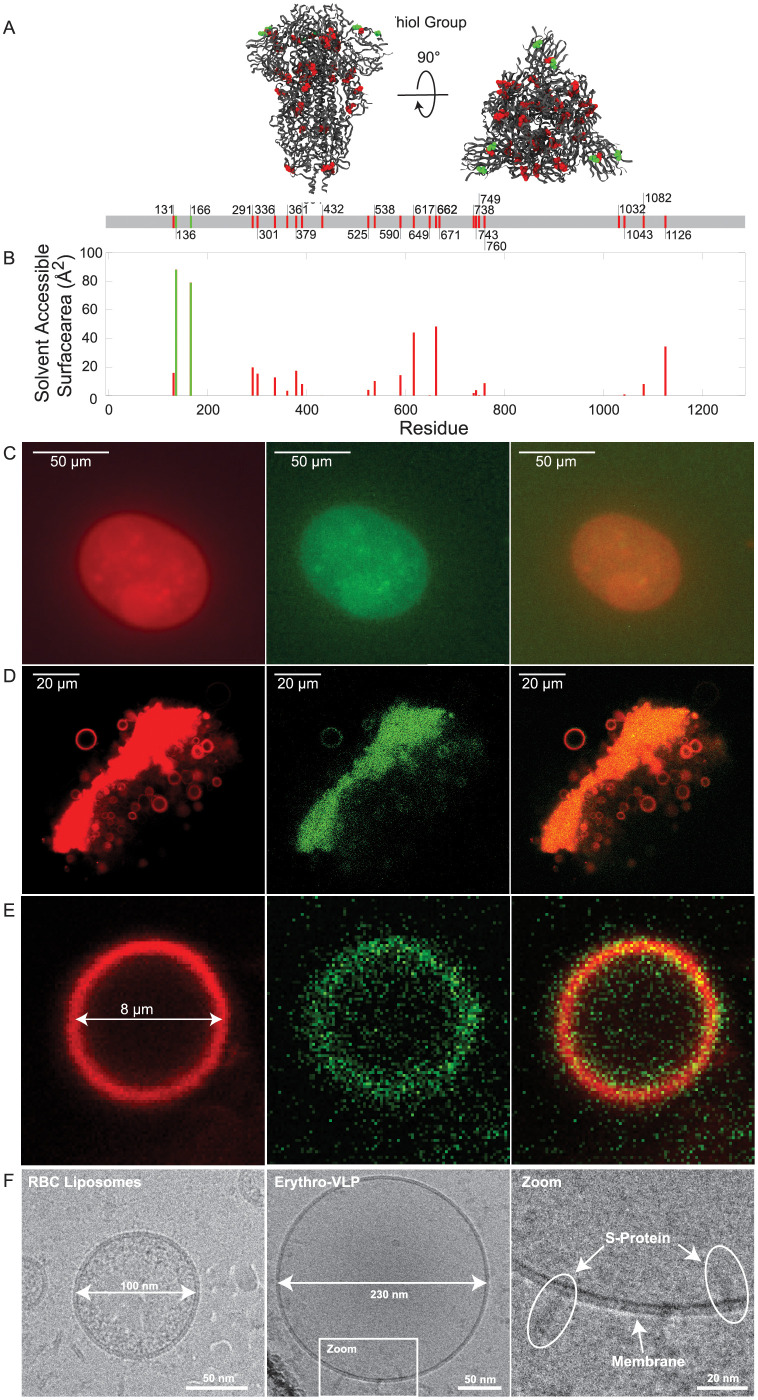
**A**: Protein structure of the SARS-CoV-2 S-protein. The protein is shown as ribbon diagram and cysteine is shown as sphere. The red and green color indicates solvent accessible and non-accessible cysteine residues. The Solvent Accessible Surface Area (SASA) was determined by the Getarea software and is graphed in **B**. **C**: Epifluorescent microscopy images of giant Erythro-VLPs grown on agarose gel. The membrane was stained in red using TR-DHPE; the SARS-CoV-2 S-protein was stained in green using Alexa Fluor 488 maleimide. **D**: CLSM images of a cluster of giant liposomes after harvesting from the agarose. **E**: Magnified image of one isolated giant Erythro-VLP taken with CLSM. Images in C-E show the red-, green-, and combined fluorescent channel, respectively. **F**: Cryo-TEM images of erythrocyte liposomes and Erythro-VLPs. Liposome sizes of ∼100 nm and ∼230 nm agree well with the results of DLS. S-proteins with their TMD anchored in the erythrocyte membranes are observed.

The RBC liposomes were doped with TR-DHPE (red) and the S-proteins were stained using Alexa Fluor 488 maleimide (AF488, green) which binds to the thiol group of cysteine [39]. The S-protein is shown as ribbon diagram in Fig 3A with the cysteine groups highlighted as red and green spheres. The solvent accessible surface area (SASA) for each of the cysteine residues is shown in Fig 3B). Following this analysis, only two cysteine residues (136 and 166) per chain are directly accessible for staining with AF488 (green bars in Fig 3B)). Thus, each protein is expected to be labeled with 6 AF488 molecules at most, as marked by the green spheres in Fig 3A.

The giant Erythro-VLPs were imaged using a combination of epifluorescent and confocal laser scanning microscopy (CLSM), shown in Fig 3C. The image was taken on the agarose gel, before harvesting the lipsomes. The liposomes appear as spherical orange objects with sizes of ∼50 *μ*m; however, such large liposomes were no longer observed after harvesting, likely due to shear stress induced damage during harvesting. The harvested liposomes show typical sizes between ∼5–10 *μ*m and were investigated using CLSM, as depicted in Fig 3D. Fig 3E shows one representative liposome in magnification. Separate imaging of the green (excitation: 488 nm) and red (excitation: 561 nm) channels shows that the S-proteins are located in the membranes (within the resolution of the microscope). The orange color is the result of the superposition of the red and green dyes and the images indicate a uniform distribution of the S-proteins in the erythrocyte membranes. A liposome with a diameter of 8 *μ*m has an estimated surface area of 800 *μ*m^2^. Given the calculated average protein density of ∼300 proteins/*μ*m^2^, each giant Erythro-VLP contained approximately 60,000 proteins and 1.8 ⋅ 10^6^ AF488 molecules. The additional intensity in the green channel in Fig 3E in the center and around the liposome indicates the presence of unbound S-proteins. While this could simply be the result of the purification process (using centrifugation), we can not exclude that some proteins unbind with time. The estimate of the S-protein density per liposome, which was based on a loading efficiency of 40% is, therefore, an upper limit for the number of proteins per liposomes that can be obtained using this protocol.

Erythrocyte liposomes, as well as Erythro-VLPs, were also imaged by cryo-TEM; the resulting images are shown in Fig 3F. The diameter of the liposomes aligns well with the results from the DLS-experiments with ∼100 nm for erythrocyte liposomes and ∼230 nm for the Erythro-VLPs. The high-resolution images show S-proteins anchored with their TMD in the erythrocyte cytoplasmic membrane. Based on the estimate above of ∼36 S-proteins per Erythro-VLP liposome, we expect that about 1–2 proteins should be visible on the cryo-TEM images, in good agreement with the observations. Proteins were found in two membrane orientations, facing outwards and also inwards the liposomes. This is likely a result of the preparation protocol. While one would expect outwards facing proteins when the surfactant is added to reconstitute the S-proteins, some liposomes seem to solubilize and recreate during this step and re-form as inside-out liposomes. The cryo-TEM images also showed some liposomes inside liposomes, which can form when membranes are solubilized. This surfactant-driven aggregation process is consistent with the slight asymmetry of the size distribution peak in SEC and DLS in Fig 2A and 2B, and the observation of aggregates in the surfactant-erythrocyte liposomes phase diagram in S1 Fig.

**Fig 4:**
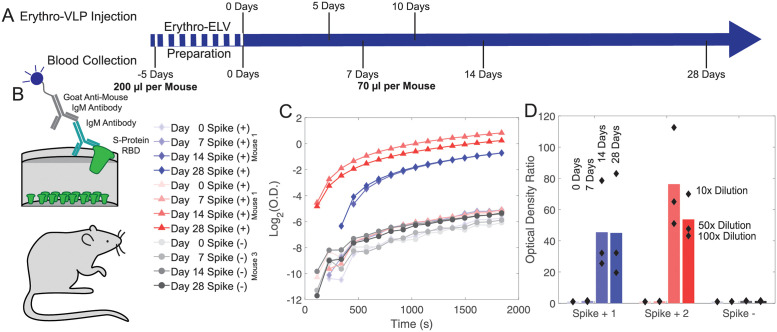
**A**: Time line of the mouse study. Each mouse received 3 injections of Erythro-VLPs suspended in sterile saline buffer at 0, 5, and 10 days. The liposome concentration in each dose was approximately 30 nM containing 8 *μ*g of the S-protein. Blood was drawn at 0 (control), 7, and 14 days. Final draw was after 28 days. **B**: An enzyme-linked immunosorbent assay (ELISA) was used for antibody detection. **C**: Optical density as function of time for the ELISA essay for all samples. **D**: Measured optical density ratios. Bars represent the mean optical density ratio averaged over all three dilution runs. Values above 1 ratio are considered positive in the SARS-CoV-2 antibody ELISA. A strong antibody response was observed in both mice after 14 days; no response was observed in the control.

A pilot mouse study was conducted over a period of 33 days involving three female mice at an age of 3 months to study a potential pharmacological effect and seroconversion *in-vivo*. The time line of the study including all injections and blood collections is displayed in Fig 4A. The mice were divided into 2 groups: Two mice received three doses of Erythro-VLPs suspended in 50 *μ*L of sterile saline buffer at days 0, 5, and 10 of the study. The liposome concentration in each dose was approximately 30 nM, containing 8 *μ*g of the S-protein. The third mouse received erythrocyte liposomes without the S-protein at an equal liposome concentration. Venous blood was collected at days 0, 7, and 28, and antibody levels were determined by ELISA (Fig 4B). Mice were immunized for the total S-protein; however, it is well documented that antibodies to the RBD are required to prevent viral entry and infection. Therefore, anti-RBD IgG antibodies were measured by ELISA. Serum was diluted 20, 50 and 100 times and absorbance values are shown as a ratio of the post-vaccination/ pre-vaccination levels in sera in Fig 4C and 4D. Since *de novo* antibody responses generally take 10 days to develop and can be low and transient in the absence of a booster dose, no signal was observed at days 0 and 7. Vaccinated mice (Mouse 1 & 2) showed a significant increase in these ratios (up to 83 and 112, respectively) on days 14 and 28 of the study. The control (Mouse 3) showed no change in the optical density throughout the samples collected.

The increased optical density in the ELISA assays 14 days after injection is a clear evidence for a successful seroconversion. Importantly, while the mice received Erythro-VLP with the full-length S-protein, antibodies to the S-protein’s RBD sub-domain were measured. This RBD is required for viral entry [40, 41]. This implies that the conformation of the S-protein in the Erythro-VLPs is not changed in such a way that the RBD domain is ‘hidden’ or modified, which is often challenging when injecting soluble proteins [15].

The Erythro-VLPs present immunopathogens to the immune system [27–29] likely when the cells are being phagocytized in the spleen. This pathway has been utilized in the past to present antigens to APCs in the spleen by attaching nanoparticles to red cells [29], and for hybrid RBC based nanovesicles [42]. An interesting point is that IgG production was triggered without an adjuvant (such as aluminium hydroxide [43, 44]), which points to some sort of a depot effect, likely related to the circulation of the Erythro-VLPs in the blood stream before they are processed in the spleen. While these first results demonstrate the potential of this pathway and the erythrocyte platform, future work is needed to elucidate its potential therapeutic use. These include, for instance, *in-vivo* toxicity evaluations and pathological analysis including vasculitis, and options for intramuscular administration.

## Conclusion

The SARS-CoV-2 Spike (S-)protein was successfully anchored in the cytoplasmic membranes of erythrocyte liposomes to create ‘Erythro-VLPs’. These ∼200 nm sized liposomes carry up to 36 S-proteins corresponding to an average protein density of ∼300 proteins/*μ*m^2^. The Erythro-VLPs, and the reconstitution of the S-proteins, were studied using Molecular Dynamics (MD) simulations, dynamic light scattering (DLS), optical microscopy and cryo-TEM. The correct insertion and functionality of the S-proteins was shown through ACE-2 binding assays. Seroconversion was observed in a mouse pilot study, and the production of antibodies was confirmed in ELISA. The results show that the Erythro-VLPs are an effective way to present the S-protein to the immune system and induce seroconversion. With a large number of similar viruses circulating in bats and camels [45], and the emergence of variants, the possibility of additional outbreaks poses major threats to global public health. The erythrocyte platform that we present in this work has therapeutic potential and can rapidly be adapted to different variants and viruses by embedding the corresponding antigenic proteins.

## Materials & methods

### Ethics approval

This research was approved by the Hamilton Integrated Research Ethics Board (HIREB) under approval number 1354-T. Informed consent was obtained from all blood donors by signing a written consent form. The authors confirm that all methods were performed in accordance with the relevant guidelines and regulations. All animal procedures for this study were approved by the McMaster University Animal Research Ethics Board (Animal Utilization Protocol 17–05-19 and Amendment #20–111 to AUP #17–05-19) in accordance with the guidelines of the Canadian Council of Animal Care.

### Immunization experiments

Three female C57BL/6J mice were obtained from the Jackson Laboratory (Bar Harbor, ME, Strain 000664), maintained in a single standard mouse cage in the same room with a constant temperature of 25°C and a 12 h light, 12 h dark cycle, and fed a control standard diet (17% kcal fat, 29% kcal protein, 54% kcal CHO, 3 kcal/g; Harlan 8640 Teklad 22/5 Rodent Diet) and provided water ad libitum. Pre-immunizaiton blood (200 *μ*L) was collected retro-orbitally in heparinized tubes. The retro-orbital bleeding of the animals was carried out under anaesthesia. RBCs were then isolated through centrifugation and washed twice using sterile saline solution and Erythro-VLPs were prepared as described. The lysing buffer was exchanged to sterile buffer saline after the preparation of red blood cell ghosts, in compliance with the approved animal utilization protocol. The mice were allowed to rest and acclimate for 5 days before immunization. Mice were immunized by injecting 50 *μ*L of Erythro-VLP in the tail vein injection and monitored daily for adverse reactions or inflammatory reactions at the injection site. Venous blood (70 *μ*L) was collected from the tail vein in heparinized tubes at days 0, 7, 14 and 28. No adverse reactions were observed.

### Preparation of small erythrocyte liposomes

The detailed protocol is described elsewhere [30, 46]. Briefly: Heparinized blood samples were collected. The blood was washed twice and the RBCs were isolated by successive centrifugation and replacing the supernatant with phosphate saline buffer (PBS). The cells were exposed to to osmotic stress by mixing hematocrit with lysis buffer (3% PBS buffer, pH 8) at a concentration of 5 vol%. The lysis buffer was pre-chilled to ∼4 °C and the reaction tube were immediately stored on ice to prevent a fast re-closing of the ruptured cells. Hemoglobin and other cellular compartments can be removed through multiple washing steps as shown in [30]. The protocol results in a white pellet containing empty erythrocyte liposomes. The resulting solution was tip sonicated 20 times for 5 s each at a power of 100 W. The reaction tube was placed on ice during sonication to prevent the sample from overheating. Afterwards, the tube was centrifuged for 15 min at ∼20,000 g. The supernatant consists of a solution of small, nanometer-sized liposomes and will be hereafter referred to as the *Blood Solution*. The protocol results in a membrane concentration of ∼14 mg/ml [30].

### Preparation of Erythro-VLPs

SARS-Cov-2 S-protein was purchased from ACROBiosystems (Product #SPN-C52H4) and was delivered in a Tris buffer (50 mM Tris (Tris(hydroxymethyl)aminomethan), 150 mM NaCl, pH 7.5 with 10% trehalose) at a concentration of 0.2 *μ*g/ml. The protein was separated in aliquots of 20 *μ*g. The cryoprotectants, glycerol and trehalose, were removed from the ACE-2 and S-proteins, respectively, by analytical SEC using a Superdex 200 increase 10/300 analytical gel filtration column (GE Healthcare). The S-protein was eluted with ultrapure H_2_O and lyophilized and resuspended by adding 50 *μ*L of the *Blood Solution*. Triton-X 100 (9002–93-1, Sigma-Aldrich) was added to achieve a concentration of 25 mM; above the critical micelle concentration (CMC) of the surfactant. The size distribution of erythrocyte liposomes at varying Triton-X 100 concentrations (in S1 Fig) shows 3 phases: Liposomes with an average diameter of 244.8±175.9 nm were observed at a Triton-X 100 concentration of 0.1 mM; below the critical micelle concentration (CMC = 0.25 mM). This liposome signal co-exists with micelles with a diameter of ∼10 nm between concentrations of 3 and 20 mM. Concentrations higher than 25 mM eventually led to an aggregation of the liposomes causing the formation of aggregates with a diameter of up to 2±0.3 *μ*m. A concentration of 25 mM was, therefore, chosen to facilitate the protein insertion as it is the upper limit of the coexistence phase of liposomes and micelles.

The sample was incubated for 3 h before adding an excess of Amberlite XAD-2 (9003–70-7, Sigma-Aldrich). These non-polar polystyrene beads are commonly used to remove surfactant, such as Triton-X 100. The sample was incubated at room temperature for 12 h. To remove potential excess Triton-X 100, which was not extracted by the beads, the supernatant containing S-protein embedded RBC membranes (Erythro-VLPs) was injected into an analytical gel filtration column and eluted with 8-fold diluted PBS. The purified fraction was then concentrated 8-fold to a working volume of 500 *μ*L (∼ 80 *μ*g/ml of total S-protein) using a Vacufuge plus from Eppendorf for subsequent BLI (Octet Red 96, ForteBio) analysis. The resulting solution will be referred to as *Erythro-VLP Solution*.

### Staining of Erythro-VLPs

The RBC membrane was fluorescently labeled by doping the bilayers with Texas Red 1,2-Dihexadecanoyl-sn-Glycero-3-Phosphoethanolamine (TR-DHPE) (Thermo Fisher, Catalog number: T1395MP). It is known to interact with liquid disordered *l*_*d*_ lipid patches and has been previously used to investigate domain formation in membranes [47, 48]. 10 mg/ml 1-Palmitoyl-2-Oleoyl-sn-Glycero-3-Phosphocholin (POPC) in chloroform was prepared containing 1 mol% TR-DHPE. POPC has been previously shown to homogenously fuse with RBC membranes [35] and facilitates the incorporation of stained lipids into the membrane. 50 *μ*L of this solution was dried in a glass vial under a constant dry nitrogen flow before adding 250 *μ*L (∼3.5 mg) of the *Blood Solution*. This solution has a concentration of TR-DHPE of 0.001 mass% and will be referred to as *Fluorescent Solution*.

After thawing, the S-protein was incubated for 20 min with a 100× excess of TCEP (Tris-(2-carboxyethyl)-phosphin). This reduces the disulfide bonds preparing the protein for staining with Alexa Fluor 488 maleimide (SCJ4600016, Sigma-Aldrich). A stock solution of 1 *μ*mol in 0.1 ml DMSO of Alexa Fluor 488 maleimide was prepared. 1 *μ*L was then added to the protein solution and incubated over night at 4°C. The protein was separated from the excess dye through centrifugation at 20,000 g for 6 h. A pellet was observed and the supernatant was replaced by fresh HEPES Buffer (20 mM Hepes, 150 mM NaCl).

The *Fluorescent Solution* was concentrated to 30 mg/ml using a Vacufuge plus from Eppendorf. The protein solution was brought to a total volume of 70 *μ*L. 5 *μ*L of the concentrated *Fluorescent Solution* was then added. Triton-X 100 (9002–93-1, Sigma-Aldrich) was added to achieve a concentration of 25 mM. The sample was incubated for 3 h before adding an excess of Amberlite XAD-2 (9003–70-7, Sigma-Aldrich) and incubating at room temperature for another 12 h.

### Preparation of giant Erythro-VLPs

Giant Erythro-VLPs were prepared using the gel assisted swelling method [31]. Briefly; Microscope cover slips were coated with a thin layer of agarose gel. Then, 12 × 1 *μ*L droplets of the Erythro-VLP solution were applied onto the gel and allowed to fully dry for ∼10 min under a nitrogen atmosphere. The glass slides were then placed in a petri dish and covered with 1 ml of growth buffer (20 mM Hepes, 150 mM NaCl, 200 mM sucrose) and incubated at room temperature for 30 min. This allows liposomes to grow on the surface of the agarose gel. Compared to the commonly known electroformation of GUV, the method produces more heterogeneous liposomes, however, has the advantage of using a saline based buffer during growth. However, it was reported to have a lower yield in isolated defect free GUV [31]. The giant Erythro-VLPs were harvested by gently pipetting ∼20 *μ*L from near the surface of the agarose and mixing it in a ratio of 1:1 with imaging buffer (20 mM Hepes, 150 mM NaCl, 200 mM glucose).

### Epi-fluorescent microscopy

Epi-fluorescent Microscopy was conducted using an Nikon Eclipse LV100 ND Microscope. The instrument is equipped with a Plan Fluor BD 10× and 20× objective with numerical apertures of 0.3 and 0.5, respectively. Images were recorded using a Nikon DS-Ri2 Camera with a resolution of 4908 × 3264 pixels and a pixel-size of 7.3 × 7.3 *μ*m. The camera is mounted via a 2.5× telescope to the microscope. All images were recorded in episcopic illumination mode using a halogen lamp. Images were recorded using the Nikon control software (NIS Elements, Version 4.60.0).

### Confocal laser scanning microscopy

Liposomes were imaged on a Nikon A1 Confocal Eclipse Ti microscope with Nikon A1plus camera. The microscope was equipped with a Plan Apo 40×/0.9 NA objective lens. Images were recorded using a resolution of 2048 × 2048 pixels and the recording speed was adjusted to ensure a optimized signal to noise ratio for each channel respectively. Two excitation modes were used: 561 nm (TR-DHPE) and 488 nm (Alexa Fluor 488 maleimide) allowing the identification of the membrane and the S-protein, respectively. The instrument was controlled by the Nikon NIS Elements software.

### Dynamic light scattering

A Zetasizer Nano ZS from Malvern Panalytical was used to determine the size distribution of the liposomes. The instrument is equipped with a 4 mW He-Ne laser (wavelength: 633 nm) and a non-invasive backscattering optics. The diffusion constant, *D*, of the liposomes is determined by DLS. This is related to the particle size via the Stokes-Einstein relation: $D=\frac{k_{B}T}{6\pi \eta r}$, where *η* is the dynamic viscosity of the solution, *k*_*B*_ is the Boltzmann constant, *T* is the sample temperature and *r* is the radius of a spherical particle. All measurements were performed at 25°C on 1 ml sample containing ∼0.5 mg/ml of membrane material.

### Biolayer interferometry (BLI)

Biotinylated human ACE-2 was purchased from Acrobiosystems (AC2-H82F9). The cryoprotectants, glycerol and trehalose, were removed from the ACE-2 proteins, respectively, by analytical SEC using a Superdex 200 increase 10/300 analytical gel filtration column (GE Healthcare). The ACE-2 protein was eluted with PBS at pH 7.4 and stored at 4°C until use.

The biotinylated human ACE-2 protein (∼11 *μ*g/ml) was immobilized onto Streptavidin (SA) biosensors (ForteBio) until a threshold of 1 nm wavelength change was reached for all sensor chips. Excess non-immobilized ACE-2 was washed off by dipping the sensor into PBS at pH 7.4 for 120 s. Subsequently, the SA biosensor was dipped into solutions of Erythro-VLPs or erythrocyte liposomes of varying doses ranging from 1× to 16× for 900 s to allow for association. Dissociation was monitored by dipping the biosensor in PBS at pH 7.4 for 900 s.

### Solvent accessible surface area (SASA) of SARS COV-2 S-protein

The SASA of the S-protein was computed through the Getarea software (http://curie.utmb.edu/getarea.html) based on the PDB ID: 6VXX S-protein structure reported by Walls and colleagues [7]. The total (backbone and sidechain) SASA was computed for all three protomers and the average and standard deviation of these three measurements are reported for each residue.

### SARS-CoV-2 ELISA

A high-throughput serological assay to identify SARS-CoV-2 antibodies in COVID-19 patients has been developed previously [49]. In brief, 384 well plates (Nunc Maxisorp, Rochester, NY, USA) were coated overnight at 4°C with 25 *μ*L/well of RBD (2 *μ*g/ml) suspended in 50 mM carbonate-bicarbonate buffer (pH 9.6). The plates were then blocked with 100 *μ*L/well of 3% skim milk prepared in PBS with 0.05% Tween 20 at room temperature for 2 h. The blocking solution was removed, and diluted mouse serum samples (1/100 prepared in 1% skim milk in PBS/0.05% Tween 20) was added to the plates for 1 h at room temperature. The plates were washed twice with PBS/0.05% Tween 20 and thrice with PBS. Bound mouse antibodies (IgG, IgA, or IgM) were detected with alkaline phosphatase conjugated goat anti-mouse IgG (*γ*-chain-specific, 1/2000, Jackson ImmunoResearch Laboratories, Inc, Westgrove, PA, USA), goat anti-mouse IgA (*α*-chain-specific; 1/500, Jackson ImmunoResearch Laboratories, Inc, Westgrove, PA, USA) antibody, or goat anti-mouse IgM (*γ*-chain-specific; 1/1000, Jackson ImmunoResearch Laboratories, Inc, Westgrove, PA, USA) antibody prepared in PBS/0.05% Tween 20. Plates were washed as before and followed with the addition of 50 *μ*L substrate (4-nitrophenylphosphate disodium salt hexahydrate in diethanolamine; MilliporeSigma, St. Louis, MO, USA). The optical density at 405 nm and 490 nm (as a reference) was measured using a BioTek 800TS microplate reader (BioTek, Winooski, VT, USA).

Values are represented as a ratio of of the observed optical density after 1840 s to the determined optical density at day 0. This value will be referred as *optical density ratio*. Values above 1 ratio are considered positive in the SARS-CoV-2 antibody ELISA.

### Molecular dynamics simulations

MD simulations were performed on a GPU accelerated computer workstation using GROMACS Version 5.1.4. The device is equipped with a 40 Core central processing unit (CPU, Intel(R) Xeon(R) CPU E5–2630 v4 @ 2.20GHz), 130 GB random-access memory (RAM) and three graphic processing units (GPU, 2 × NVIDIA 1080 TDI + 1 × GeForce GT 730) [35]. A total of 4 simulation systems were created. All-atom simulations can provide a detailed insight into membrane structure and dynamics but are limited to small systems due to the increased computation time [50–52]. Coarse grained models are thus used to study large-scale membrane and protein dynamics. First, a coarse grained model of a single S-protein was created using the CHARMM-GUI Martini Solution-builder (http://charmm-gui.org/) [53]. The all-atom S-protein model from [34] which bases on the RBD-down protein structure (PDB database: 6VXX) was used as input for CHARMM-GUI. Model 1 (see [34] for naming convention) was used for the heptad repeat linker 2 (HR2), the transmembrane-domain (HR2-TM) and the CPD. The System was charge-neutralized by adding Na^+^ and Cl^−^ counter-ions. A second model was created by first removing water from the simulation box and adding Triton-X 100 molecules to archive a concentration of 25 mM. The system was re-dissolved with MARTINI water and neutralizing ions were added. Two membrane-S-protein complexes containing one SARS-CoV-2 S-protein, a RBC membrane mimic respectively were designed using the CHARMM-GUI membrane-builder (http://charmm-gui.org/) [53]. The bilayer composition was chosen to match the lipid concentrations of a RBC membrane as has been shown previously [35]. In one model, the protein’s TMD was embedded into the membrane. For the second model, the CPD was placed in close proximity to the membrane mimic. Triton-X 100 at a concentration of 25 mM was added to both simulations. All models are available from the authors upon request. All models were energy-minimized using steepest descent and equilibrated for 5 ns in the NPT ensemble (constant pressure and temperature). A short range van der Waal cutoff of 1.1 nm and a potential-shift-verlet coulomb modifier were used and periodic boundary conditions were applied to all three dimensions. Neighbor lists were updated in intervals of 20 steps. The temperature was coupled through a v-rescale thermostat at a constant pressure of 1 bar using Parrinello-Rahman semi-isotropic weak coupling (*τ* = 12 ps; compressibility *β* = 3 ⋅ 10^−4^ bar^−1^). All simulations were run for a total of 500 ns. The model containing a single S-protein in close proximity to the membrane mimic was first run for 500 ns with position constraints applied to the protein in all spacial direction. The constraints were removed and the system was simulated for additional 500 ns.

The GROMACS built-in function *gmx angle* was used to calculate the tilt angle Θ indicated in Fig 1D. Density maps were calculated using the GROMACS built-in function *gmx densmap*.

The GROMACS configuration files of all simulations are provided as *Supporting Information*.

### ELV-protein co-sedimentation assay

The S-protein loading efficiency onto the Erythro-VLP was determined using UV–visible light spectroscopy using a Nanophotometer NP80 from IMPLEN. Erythro-VLP carrying Alexa-fluor 488 labeled S-protein were prepared as described above. A UV-vis spectra was measured and the the sample was incubated for 12 h with an excess of Amberlite XAD-2 (9003–70-7, Sigma-Aldrich). The samples were centrifuged for 2 h and a UV-vis spectrum was measured. Both spectra are graphed in S2 Fig. A peak at 488 nm was observed in both samples resulting from the stained proteins. The signal was observed to decrease by 40%. This decrease is assumed to be the result of sedimented liposomes and we consequently estimate a loading efficiency of ∼40%.

### Determining the Triton-X 100—Erythrocyte liposome phase diagram

Erythrocyte liposomes where first prepared according to the protocol described above. 10 samples, 1 ml each, containing ∼0.5 mg/ml of membrane material were prepared with Triton-X 100 at concentrations of 0.1, 1, 3, 5, 10, 15, 20, 25, 30, 35 mM. The size distribution of the liposomes and Triton-X 100 micelles were then determined using a Zetasizer Nano ZS from Malvern Panalytical. All measurements were performed at 25°C.

### Cryo-TEM

Sample vitrification for cryo-TEM was performed using a Vitrobot Mark IV (Thermo Fisher Scientific). Before samples were applied to the EM grids (C-flat 2/2–2Cu-T), grids were washed with chloroform for 2 h and treated with negative glow discharge in air at 5 mA for 15 s. For all samples, a volume of 3.6 *μ*L was applied to the holey carbon grids and manually blotted using the Vitrobot blotting paper (Standard Vitrobot Filter Paper, ⌀55/20 mm, Grade 595). Right after blotting, a new drop of the sample was applied to the EM grid and blotted again using the standard routine with the two blotting pads in the Vitrobot Mark IV (Thermo Fisher Scientific) for 3 s and with a blot force + 1 before they were plunged into liquid ethane. The Vitrobot was set at 25°C and 100% relative humidity. Data acquisition was performed at the Facility for Electron Microscopy Research (FEMR) at McGill University using a FEI Tecnai G2 F20 200 kV microscope equipped with TVIPS TemCam XF416(ES) 16 MP CMOS camera System. Grids were loaded into the microscope using a Gatan 626 single-tilt cryo-holder and were imaged using SerialEM software. Images were collected at a magnification of 62,000×, which produced images with a calibrated pixel size of 1.761 Å. Images were collected with a total dose of ∼50 e^−^/Å^−2^ using a defocus ranging from −2 *μ*m to −2.50 *μ*m. Images were cropped and prepared using Adobe Illustrator.

## Supporting information

S1 Fig DLS size distribution of erythrocyte liposomes in aqueous solution and Triton-X 100 concentrations ranging from 0.1 mM to 35 mM. A single distribution 244.8±175.9 nm resulting from liposomes was observed at a Triton-X 100 concentration of 0.1 mM; below the critical micelle concentration (CMC = 0.25 mM). This liposome signal co-exists with micelles with a diameter of 10 nm at concentrations from 3 mM to 20 mM. Concentrations higher than 20 mM eventually lead to an aggregation of the liposomes to form aggregates with a diameter of up to 2±0.3 *μ*m.
(JPG)

S2 Fig UV-vis spectra of Erythro-VLP carrying Alexa Fluor 488 maleimide tagged S-protein before and after incubation. (JPG)

S3 Fig Biolayer interferometry analysis of the binding of erythrocyte liposomes to the human ACE-2 receptor. Association and dissociation curves for the binding of various concentrations of erythrocyte liposomes to the human ACE-2 receptor is shown in accordance with the color coding in the figure inset. Control association and dissociation curves for erythrocyte liposomes in the absence of human ACE-2 immobilized onto the biosensor are shown in purple.
(JPG)

S1 Table ELISA assay: Measured optical densities. (XLSX)

S1 File Coarse grained molecular structure file representing a single S-Protein in close proximity to the membrane with Triton-X100. (GRO)

S2 File Coarse grained molecular structure file representing a single S-Protein embedded into the membrane with Triton-X100. (GRO)

S3 File Coarse grained molecular structure file representing a single S-Protein in an aqueous solution. (GRO)

S4 File Coarse grained molecular structure file representing a single S-Protein in an aqueous solution and Triton-X100. (GRO)
