# Supplementary figures and images for: Erythro-VLPs: Anchoring SARS-CoV-2 spike proteins in erythrocyte liposomes
Source: PLoS One. 2022 Mar 11;17(3):e0263671. doi: 10.1371/journal.pone.0263671 (PMC8916654; doi:10.1371/journal.pone.0263671)

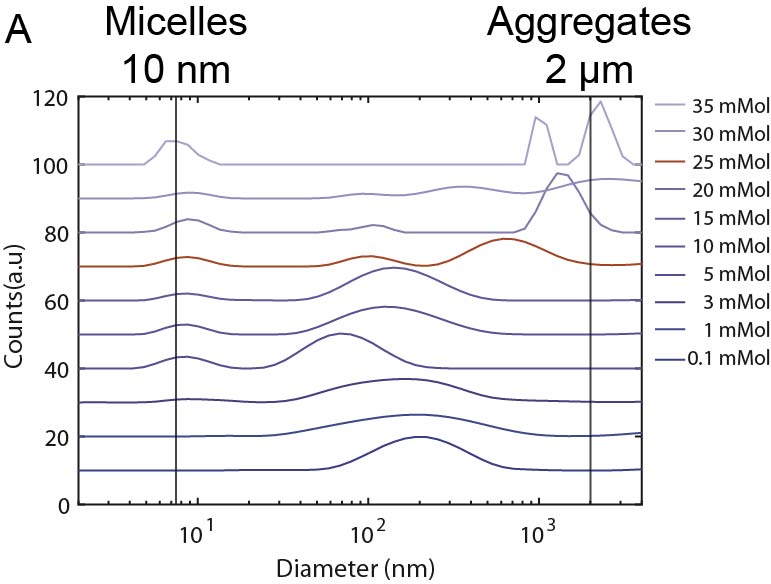

Supplement: S1 Fig — A single distribution 244.8±175.9 nm resulting from liposomes was observed at a Triton-X 100 concentration of 0.1 mM; below the critical micelle concentration (CMC = 0.25 mM). This liposome signal co-exists with micelles with a diameter of 10 nm at concentrations from 3 mM to 20 mM. Concentrations higher than 20 mM eventually lead to an aggregation of the liposomes to form aggregates with a diameter of up to 2±0.3 μm. (JPG) [file pone.0263671.s001.jpg]

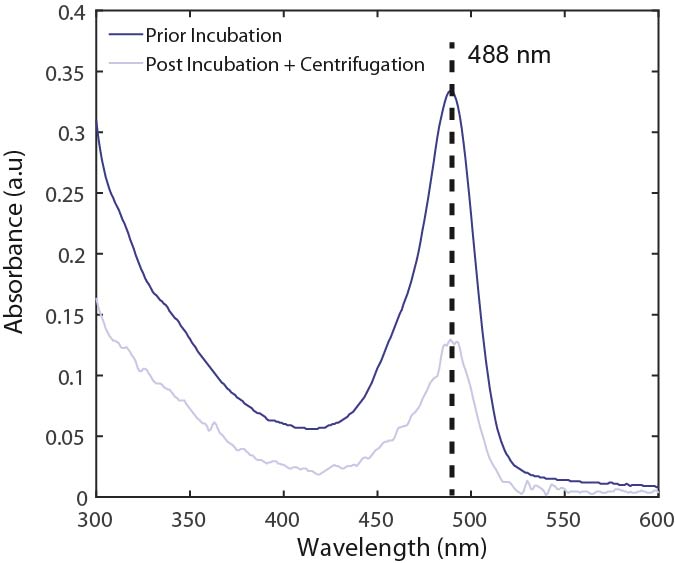

Supplement: S2 Fig — (JPG) [file pone.0263671.s002.jpg]

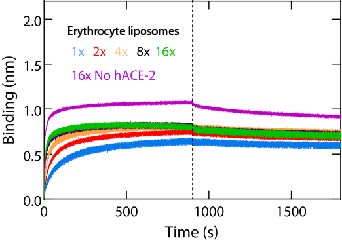

Supplement: S3 Fig — Association and dissociation curves for the binding of various concentrations of erythrocyte liposomes to the human ACE-2 receptor is shown in accordance with the color coding in the figure inset. Control association and dissociation curves for erythrocyte liposomes in the absence of human ACE-2 immobilized onto the biosensor are shown in purple. (JPG) [file pone.0263671.s003.jpg]
